# Dr. James McIlroy

**Published:** 1912-08-17

**Authors:** 


### Obituary.

Dr. James McIlroy, of Ballycastle, co. Antrim, has died after a short illness at his residence, in his sixty-eighth year. Dr. McIlroy was formerly in practice at Lavan, and was dispensing medical officer there for over twenty years. Subsequently he held a similar appointment in the Ballycastle district, and was also appointed medical officer to the workhouse. He was a Justice of the Peace for the county of Antrim, and medical officer to the Royal Irish Constabulary and the Rathlin Lighthouse. It is noteworthy that two of his four daughters have followed their father's profession—one having attained a wide reputation in obstetrics and gynæcology, the other being a well-known ophthalmic surgeon, both practising in Glasgow.

